# Diagnostic methods to cutaneous leishmaniasis detection in domestic dogs and cats*

**DOI:** 10.1590/abd1806-4841.20153716

**Published:** 2015

**Authors:** Daliah Alves Coelho Trevisan, Maria Valdrinez Campana Lonardoni, Izabel Galhardo Demarchi

**Affiliations:** 1 Universidade Estadual de Maringá - Maringá (PR), Brazil

**Keywords:** Americas, *Leishmania*, Leishmaniasis, cutaneous, Sensitivity and specificity

## Abstract

Cutaneous leishmaniasis is caused by different species of *Leishmania*. In domestic animals such as dogs and cats, the diagnostic consists of clinical, epidemiological and serological tests, which changes among countries all around the world. Because of this diversity in the methods selected, we propose this systematic literature review to identify the methods of laboratory diagnosis used to detect cutaneous leishmaniasis in domestic dogs and cats in the Americas. Articles published in the last 5 years were searched in PubMed, ISI Web of Science, LILACS and Scielo, and we selected 10 papers about cutaneous leishmaniasis in dogs and cats in the Americas. In Brazil, often the indirect immunofluorescence and enzyme immunoassay (ELISA) have been applied. Other countries like United States and Mexico have been using antigenic fractions for antibodies detections by Western blot. ELISA and Western blot showed a higher sensitivity and efficacy in the detection of leishmaniasis. Analysis of sensibility and specificity of the methods was rarely used. Although confirmatory to leishmaniasis, direct methods for parasites detection and polymerase chain reaction showed low positivity in disease detection. We suggested that more than one method should be used for the detection of feline and canine leishmaniasis. Serological methods such as Western blot and enzyme immunoassay have a high efficacy in the diagnosis of this disease.

## INTRODUCTION

American cutaneous leishmaniasis (ACL) is caused by protozoa of the genus *Leishmania*. The parasite transmission cycle occurs between a phlebotomine sandfly (vector) and wild animals. Men and domestic animals such as dogs and cats can become infected when penetrating in this ecosystem. The infection is characterized by lesions of skin and mucous membranes.^1^

Currently there are 21 known species of *Leishmania* that cause diseases in humans and 12 species that infect animals. In the Americas, main species involved in cutaneous leishmaniasis in pets are *Leishmania (Viannia) braziliensis, Leishmania (Leishmania) amazonensis*, and, in the Old World, the species *Leishmania infantum.* ^1,2,3^ There are many records of cases of cutaneous leishmaniasis in domestic cats and dogs, but as there is no scientific evidence that these animals are natural hosts of this parasite, they are still considered accidental reservoirs.^1,4,5^

Leishmaniasis in domestic animals has a higher incidence in Europe and South America. The number of cases of the disease in domestic animals is increasing, which values the diagnosis to confirm the disease and prevent its spread. Usually, diagnosis of leishmaniasis in humans and animals consists of clinical, epidemiological and laboratory tests. Laboratory tests recommended for the diagnosis of cutaneous leishmaniasis are: parasitological (direct collection of lesion material), immunological (antibody, antigen or cellular immune response tests), and molecular, such as polymerase chain reaction (PCR), among others.^1,2,3,6^

In Brazil, seropositive and/or parasitological positive are euthanized based on Resolution No. 714 of June 20th, 2002, from the Federal Council of Veterinary Medicine, which sets forth the procedures and methods of euthanasia in animals, among other provisions. This practice is criticized by the pet owners, some veterinarians and researchers. In some countries, such as Brazil, there are other recommendations as control of reservoirs, diagnosis and measures to prevent contamination of healthy dogs.^7^

In 2011, Bill No. 1738/11 was proposed in Brazil, which recommends not to euthanize animals with confirmed leishmaniasis. Euthanasia of animals carrying the disease would be the best way from a health point of view, but the costs with capture, tests and euthanasia of the animals could be reversed to combat the vectors that transmit the disease.^8^

Epidemiology, immunopathogenesis and laboratory diagnosis of leishmaniasis (cutaneous and visceral) in domestic animals have been widely studied in several regions of the world. However, most of the studies address methodologies for diagnosis of canine visceral leishmaniasis and just a few discusses cutaneous and mucocutaneous forms. Furthermore, diagnostic techniques vary between countries around the world, which can change the sensitivity, specificity and efficacy of the tests.^2,9-17^ Therefore, we propose this review with the objective to identify and characterize laboratory diagnostic methods used for the detection of cutaneous leishmaniasis in domestic dogs and cats in the Americas in order to analyze the sensitivity and specificity of the tests and indicate the most efficient ones, which should be used for the detection and monitoring of leishmaniasis in these animals.

## METHODS

This is a systematic review, in which the types of eligible studies were original articles, master dissertations, doctoral theses and book chapters published in the last five years and available. Only articles in Portuguese, English and Spanish were considered for the analysis.

### Search strategy

Search strategy in PubMed (US National Library of Medicine) consisted in using MeSH database for the terms "leishmaniasis", "cutaneous leishmaniasis", "mucocutaneous leishmaniasis", "diffuse cutaneous leishmaniasis", "*Leishmania*", "sensitivity and specificity", "animals", "diagnosis", "cat diseases", "cats", "dog diseases", "dogs". We also searched these terms and free terms in LILACS (Latin American and Caribbean Health Sciences Information Center), ISI Web of Science and SciELO database. Terms used in LILACS were: leishmaniasis, *Leishmania*, and diagnostic sensitivity and specificity.

### Data extraction

Data were collected from August to September 2013. The potentially relevant studies were selected by the analysis of abstracts. Copies of full text of all studies identified as potentially relevant were retrieved. From 254 potential studies, 10 on cutaneous leishmaniasis in domestic animals of the Americas were selected. The remaining articles addressed visceral leishmaniasis, leishmaniasis in the Old World, leishmaniasis in humans, or they were literature reviews. Doctoral theses, master dissertations or book chapterswere not found.

## RESULTS AND DISCUSSION

We analyzed 10 articles, seven on leishmaniasis in dogs, two on leishmaniasis in cats and one on both.^10,11,13,18-24^ Laboratory tests most commonly used in the Americas for the diagnosis of cutaneous leishmaniasis in these animals were: detection of anti-*Leishmania* antibodies by indirect immunofluorescence (IIF) and enzyme immunoassay (ELISA), antigens research using Western blot technique and molecular techniques such as polymerase chain reaction (PCR) (Tables 1 and 2).

**Table 1:** Characteristics of studies included in the review on leishmaniasis in domestic dogs

Study	Country	Animals (n)	*Leishmania* species	Sample / Diagnostic Test	Test reactivity
				Lesion material/ direct test	Positive
					
Cavalcanti et al, 2012^18^	Brazil	1	*L. infantum*	Blood/culture	Reagent
				PCR*	Positive
				ELISA**	Title 640
					
				Lesion material/Histological	Positive in one animal
					
Figueiredo et al, 2009^19^	Brazil	177	*L. braziliensis*	Blood/ IIF***	Reactivity of 10% (titles of 40 to 320)
				ELISA	Reactivity in 10.7%
					
Heusser-Junior et al, 2010^20^	Brazil	275	*L. (V.) braziliensis*	Lesion material/ direct test	Negative
				Blood/IIF	1.1% (3 dogs)
				ELISA	1.4% (4 dogs)
					
				Cellular / intradermal test	1.8% (5 dogs)
					Negative
					Negative
					
Massunari, et al, 2009^11^	Brazil	146	*L. (V) braziliensis*	Lesion material/ direct test	17.1% (25 animals)
				PCR	2 dogs
				Blood/IIF	Negative
				PCR	
				Culture	
					
				Bone marrow/culture	Negative
				PCR	Negative
					
Soccol et al, 2009^21^	Brazil	31	*L. braziliensis*	Tissue / Histological	Negative
				Blood/ELISA	29% (9 animals)

* PCR: polymerase chain reaction;

** ELISA: enzyme immunoassay;

*** IIF: indirect immunofluorescence.

**Table 2:** Characteristics of articles included in the review of cutaneous leishmaniasis in domestic dogs, except laboratory results

Study	Country	Animals (n)	*Leishmania* species	Sample / Diagnostic Test
Longoni et al, 2011^10^	Mexico	70	*L. mexicana*	Blood/ELISA**
			*L. braziliensis*	Western Blot
			*L. panamensis*	
				
Lópes-Céspedes et al, 2012^22^	Mexico	412	*L. mexicana*	Blood/ELISA**
			*L. braziliensis*	Western Blot
			*L infantum*	Culture
				
Oliveira, et al, 2011^23^	Brazil	26	*L. (V.) braziliensis*	
				Lesion material/ direct test
				Blood/PCR*

* PCR: polymerase chain reaction;

** ELISA: enzyme immunoassay.

In Brazil and Mexico, the most widely used tests for the diagnosis of canine cutaneous leishmaniasis are the direct test of the lesion material, IIF, ELISA, PCR and culture (Tables 1 and 2). Of these, IIF testing and enzyme immunoassay were those with higher reactivity when animal blood was used, being considered the most sensitive for the diagnosis of leishmaniasis in dogs. According to the Ministry of Health (BRAZIL, 2007),^1^ on cutaneous leishmaniasis, the tests of choice for diagnosis in dogs are direct examination and serological (IIF and ELISA).

Longoni et al (2011)^10^ used Fe-SODe (iron superoxide dismutase) and H (total extract of *Leishmania* culture) antigenic fractions for the reaction of enzyme immunoassay and Western blot. These fractions were extracted and purified from parasites culture of *L.mexicana, L. braziliensis* and *L. panamensis* species. Of the 70 dogs, only four (2.8%) were positive in the ELISA H-fraction, one for *L. mexicana*, and 3 for *L. braziliensis*. Fe-SODe ELISA-fraction was positive in 37 animals (25.9%), identifying mainly *L. mexicana* and *L. braziliensis*, and just a few *L. panamensis*. Researchers also used Fe-SODe fraction in the research for the presence of antibodies by Western blot method, in which they achieved 33 (47.1%) positive results for *L. mexicana* and 30 (42.9%) for *L. braziliensis*. No sample was tested in Western blot for *L. panamensis*, because the enzyme immunoassay didn't show a good sensitivity.

Lópes-Céspedes et al (2012)^22^ used H and Fe-SODe fractions of *L.mexicana, L. braziliensis* and *L. infantum* parasite cultures for serological testing by enzyme immunoassay and Western blot technique, similar to Longoni et al (2011)^10^ *.* The study analyzed samples from 412 dogs; ELISA-H had low sensitivity, and Fe-SODe-ELISA and Western blot Fe-SODe were more sensitive to all species investigated. Positivity for *L.braziliensis* was 7.5%, for *L. mexicana* was 20.6%, and for *L.infantum* was 6.1%. The concordance of ELISA and Western blot using Fe-SODe fraction was greater than 84%, reaching up to 99%.

Use of antigenic Fe-SODe fraction based on Western blot is an optimal method for the detection of both feline and canine leishmaniasis.^10,13,22^

Oliveira et al (2011)^23^, analyzed 26 samples from different regions of northwestern Paraná (Brazil): collection of lesion material from dogs for direct research and PCR was performed in eight, and blood from 18 animals was collected for PCR. Researchers tested the following primers: MP34-MP1L (best performance), B1-B2, LU5A-LB3C, LBF1-LBR1 and 13A-13B. For the lesion material, MP34-MP1L primer had 100% positivity and, for the blood, the positivity was 83.3%. In the direct test, no sample was positive.

In Brazil and USA, tests for the diagnosis of leishmaniasis in cats were histopathological examination of the lesion, IIF, ELISA and PCR. As with dogs, the test that showed greater positivity in detection of leishmaniasis was enzyme immunoassay (Table 3).

**Table 3:** Characteristics of articles included in the review of cutaneous leishmaniasis in domestic cats

Study	Country	Animals (n)	Leishmania species	Sample / Diagnostic Test	Test reactivity
Figueiredo, et al 2009^19^	Brazil	43	L. braziliensis	Lesion material/Histological	Negative
				Blood/IIF***	Non-reagent 2.4%
				ELISA**	
					
Trainoret al 2010^24^	USA	8	L. mexicana amazonensis	Lesion material/Histological	Negative
				PCR*	62.5% (5)

* PCR: polymerase chain reaction;

** ELISA: enzyme immunoassay;

*** IIF: indirect immunofluorescence.

Longoni et al (2012)^13^, using antigenic fractions (H and Fe-SODe) of cultures of *L. mexicana, L. braziliensis* and *L. Infantum*, investigated the presence of antibodies in the serum of cats using ELISA and Western blot techniques. Samples from 96 cats were analyzed in ELISA-H test, 13.68% were positive for *L. infantum*, 5.26% for *L. braziliensis* and only one sample was positive for *L. mexicana*. In ELISA Fe-SODe, positivity was higher (22.10%) for *L. infantum*, reaching 10.25% for *L. mexicana* and 11.57% for *L. braziliensis*. In Western blot Fe-SODe test, 10.5% of the samples were positive for both *L. mexicana* and *L. braziliensis*, and 20% were positive for *L. infantum*.

Regarding testing and *Leishmania* species investigated in the selected studies in this review, in Brazil, most studies performed the research for antibodies or *L. braziliensis* antigens in both dogs and cats. This can be justified, since according to the Ministry of Health^1^, the main species causing the disease in this country is *L. braziliensis*. In the study of Longoni et al (2011)^10^, in the region of Tulum (Mexico), the most present specie was *L. mexicana*. On the other hand, in the region of Celestu'n (Mexico), it was reported more cases of infection with *L. braziliensis.* Lópes-Céspedes et al (2012)^22^ and Oliveira et al (2011)^23^ detected a higher number of infections caused by *L. braziliensis*.

Of the 10 studies analyzed in this review, only two assessed the sensitivity and specificity of the methods used. Few studies have used these quality parameters, which are important since they show the test's ability to detect truly positive and negative cases, excluding possible false positives and/or false negatives.^25^

In the study of Lópes-Céspedes et al(2012), for dogs, the sensitivity obtained by ELISA and Western blot Fe-SODe was 96.2% for *L. braziliensis* and 100% for *L. infantum* and *L. mexicana*.^22^Specificity was above 99% for all species. In the study of Longoni et al (2012), for cats, the sensitivity of ELISA and Western blot using Fe-SODe fraction was 100%, with specificity between 97% and 100%, a positive predictive value between 91% and 100%, and a negative predictive value of 100%.^13^

The studies that used more than one method for the diagnosis of leishmaniasis showed high concordance between tests, and the conduction of more than one method supports a more accurate and secure diagnosis of leishmaniasis. A study by Oswaldo Cruz Foundation (Fiocruz), in Brazil, suggested that the diagnosis of canine visceral leishmaniasis is most effective when used in combined tests. Thus, there is a decrease in false negative results as well as false positives.^26^

Brito (1999), in Pernambuco, using samples from humans, compared the sensitivity and specificity of IIF and ELISA tests with Western blot containing significant antigen of *L.braziliensis*.^27^ IIF and ELISA techniques presented a sensitivity of 51.7% and 62.1%, and specificity of 78.6% and 71.4%, respectively. In this case it was not observed difference between the methods. However, comparing it with the results of Western blot, sensitivity was 90.9% and specificity was 100%, higher than IIF and ELISA. This study showed that the use of antigenic fractions to research the presence of antibodies by Western blot technique is much more sensitive for the detection of leishmaniasis.

## CONCLUSION

The most used diagnostic methods to detect canine and feline cutaneous leishmaniasis are indirect immunofluorescence (IIF), enzyme immunoassay, polymerase chain reaction (PCR), direct tests of the lesion material, and Western blot. Western blot technique from purified Fe-SODe fraction showed satisfactory results, with high sensitivity, specificity and efficacy for the detection of this disease in animals. We suggest that more than one technique should be used for detection of feline and canine cutaneous leishmaniasis and that Western blot technique using Fe-SODe fraction should be widely used.
